# Supplementary material for: Identification of photoperiod-induced specific miRNAs in the adrenal glands of Sunite sheep (Ovis aries)
Source: Front Vet Sci. 2022 Jul 22;9:888207. doi: 10.3389/fvets.2022.888207 (PMC9354845; doi:10.3389/fvets.2022.888207)
Supplement: Supplementary file 1 [file Data_Sheet_1.ZIP › Supplementary file/miRDeep2 file.zip!/miRDeep2 file/Know_Structure/SPLP42A2/oar-mir-3958.pdf]

miRBase precursor : oar-mir-3958
Total read count : 11828
oar-miR-3958-5p read count : 17
oar-miR-3958-3p read count : 11808
remaining reads : 3

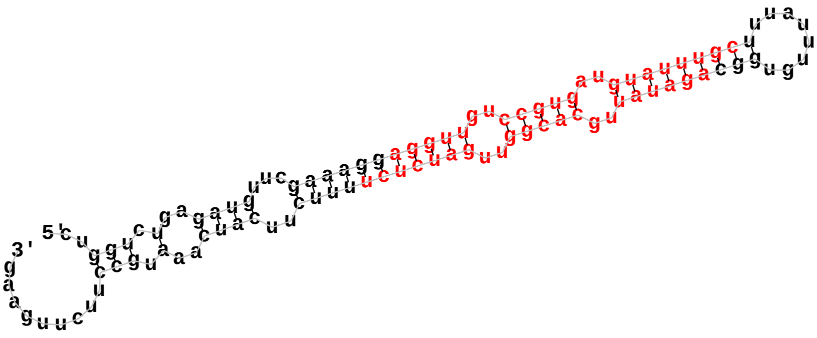

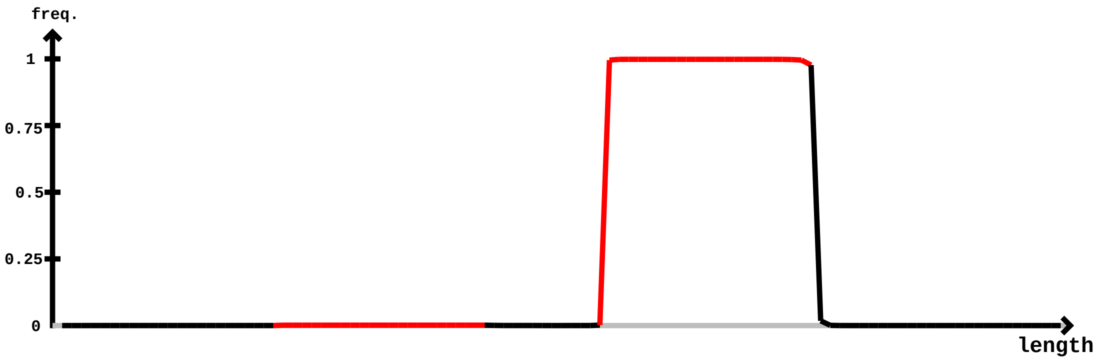


```
                                                                   oar-miR-3958-3p
                                oar-miR-3958-5p
5'- cuggucugagauguucgaaaggagguuguccgugauguauuugcuuuauuuguggcagauauugcacgguugaucucuuuucuucaucaaaugccuucuugaag   -3'   exp
    ..(((.(..((((...((((((((((..(((((..(((((((((.........)))))))))..)))))..))))))))))..))))..).)))..........   reads   mm   sample
    ..ggucugagauguucgaaagg..................................................................................   1       0    seq
    ......................agguuguccgugauguauuug.............................................................   2       0    seq
    ......................agguuguccgugauguauuugc............................................................   8       0    seq
    ......................agguuguccgugauguauuugcu...........................................................   6       0    seq
    ...........................guccgugauguauuugcuuuau.......................................................   1       0    seq
    ............................uccgugauguauuugcuuuauu......................................................   1       0    seq
    ....................................................uggcagauauugcacgguugauc.............................   1       0    seq
    .......................................................cagauauugcacgguugaucuc...........................   1       0    seq
    .......................................................cagauauugcacgguugaucucu..........................   18      0    seq
    ........................................................agauauugcacgguugau..............................   2       0    seq
    ........................................................agauauugcacgguugauc.............................   8       0    seq
    ........................................................agauauugcacgguugaucu............................   23      0    seq
    ........................................................agauauugcacgguugaucuc...........................   222     0    seq
    ........................................................agauauugcacgguugaucucu..........................   11287   0    seq
    ........................................................agauauugcacgguugaucucuu.........................   202     0    seq
    ........................................................agauauugcacgguugaucucuuu........................   8       0    seq
    ........................................................agauauugcacgguugaucucuuuu.......................   1       0    seq
    .........................................................gauauugcacgguugaucucu..........................   34      0    seq
    ..........................................................auauugcacgguugaucucu..........................   2       0    seq
```
